# Contrasting anthropogenic drivers of mercury bioaccumulation in fish and associated dietary exposure risks in Amazon and Cerrado floodplain lakes

**DOI:** 10.1007/s10653-026-03335-0

**Published:** 2026-07-14

**Authors:** Lucas Cabrera Monteiro, Ludgero Cardoso Galli Vieira, José Vicente Elias Bernardi, Ronaldo de Almeida, Cássio da Silva Cabral, Fabrício Barreto Teresa, João Carlos Nabout, Wanderley Rodrigues Bastos

**Affiliations:** 1 Programa de Pós-Graduação em Ecologia, Instituto de Ciências Biológicas Universidade de Brasília, Brasília, Distrito Federal Brazil; 2 Núcleo de Estudos e Pesquisas Ambientais e Limnológicas, Faculdade UnB Planaltina, Universidade de Brasília, Brasília, Distrito Federal Brazil; 3 Present Address: Laboratório de Geoestatística e Geodésia, Faculdade UnB Planaltina, Universidade de Brasília, Brasília, Distrito Federal Brazil; 4 Laboratório de Biogeoquímica Ambiental WCP, Universidade Federal de Rondônia, Porto Velho, Rondônia Brazil; 5 Laboratório de Biogeografia e Ecologia Aquática, Universidade Estadual de Goiás, Anápolis, Goiás Brazil

**Keywords:** Artisanal and small-scale gold mining, Biomagnification, Deforestation, Floodplain, Mercury exposure

## Abstract

We evaluated total mercury (THg) concentrations in fish from lakes in two major South American river basins: the Madeira River basin in the Western Amazon, impacted by gold mining, deforestation, and wildfires; and the Araguaia River basin in the Brazilian Savanna (Cerrado), where deforestation and wildfires are the main anthropogenic pressures. Total Hg concentrations ranged from 37 to 2473 µg kg^−1^ in Puruzinho Lake (Madeira River basin) and from 6 to 1947 µg kg^−1^ in floodplain lakes of the Araguaia River basin. Comparisons between river basins revealed significantly higher THg concentrations in the Madeira River basin for detritivorous, carnivorous, and piscivorous fish, whereas no significant difference was observed for planktivorous species. Within both basins, THg concentrations in planktivorous, carnivorous and piscivorous fish were significantly higher than those observed in detritivores. The trophic magnification slope further confirmed Hg biomagnification along the food web, with a higher coefficient of determination in the Araguaia River basin. Estimated weekly intake calculations indicated substantially greater dietary Hg exposure in the Madeira basin under moderate to high fish consumption scenarios, particularly for predatory species. Although Hg concentrations and the associated risks from fish consumption are higher in the Madeira River basin, which has a long history of gold mining, our results highlight land-use change as an important driver of Hg bioaccumulation in fish across Neotropical ecosystems.

**Supplementary Information:**

The online version contains supplementary material available at 10.1007/s10653-026-03335-0.

## Introduction

Mercury (Hg) is recognized as a priority global pollutant due to its high toxicity, its ability to bioaccumulate in organisms, and its biomagnification through food webs (UNEP, 2019). Mercury is naturally mobilized to surface ecosystems through rock weathering, volcanic emissions, and marine aerosols (Schneider et al., 2023). However, human activities, including artisanal and small-scale gold mining (ASGM), fossil fuel combustion, and industrial processes, currently account for most global Hg emissions (Outridge et al., 2018). In tropical regions, particularly in South America, the rapid deforestation observed in recent decades has become an additional and significant driver of Hg re-mobilization, reducing Hg stocks in vegetation and intensifying the erosion and leaching of particle-bound Hg to aquatic ecosystems (Fisher et al., 2023).

Deforestation in the Amazon and Cerrado (Brazilian Savanna), the two largest South American biomes, remains one of the dominant land-use pressures in Brazil, with both regions together accounting for more than 80% of all native vegetation loss recorded since 2019, largely driven by agricultural expansion (MapBiomas, 2025a). Fire is pervasive in both biomes, and the Cerrado consistently exhibits the highest burned area in the country, while the Amazon continues to experience extensive fire activity associated with forest degradation and illegal clearing (MapBiomas, 2025b). In the Amazon, these disturbances occur alongside long-standing ASGM, which is a major source of metallic Hg emissions (Crespo-Lopez et al., 2021). As a result, deforestation-driven soil erosion, fire-induced Hg volatilization, and mining-derived Hg inputs enhance the mobilization of inorganic Hg to aquatic ecosystems, where prevailing biogeochemical conditions can favor its conversion to methylmercury and subsequent bioaccumulation in aquatic food webs (Lacerda et al., 2012; Paiva et al., 2024).

The Madeira River is one of the most ecologically and biogeochemically complex tributaries of the Amazon Basin, supporting approximately 25% of the fish species predicted to occur across the Amazon region (Cella-Ribeiro et al., 2016). However, decades of ASGM, extensive deforestation, and the construction of run-of-river hydropower dams have profoundly altered sediment dynamics, water chemistry, and habitat connectivity (Cella-Ribeiro et al., 2017; Almeida et al., 2019; Bastos et al., 2020), with both direct and indirect pathways influencing Hg cycling and bioaccumulation in the ichthyofauna (Reuther, 1994; Lacerda et al., 2012; Bastos et al., 2015). In contrast, the Araguaia River is the main free-flowing river of Central Brazil and supports the highest fish species richness in the Cerrado biome (Latrubesse et al., 2019). The Araguaia Basin has experienced only brief and spatially restricted mining episodes (Silva, 2018; Ulrich et al., 2021), which have not resulted in significant Hg contamination footprints when compared with ASGM-affected Amazonian systems. Nevertheless, the Araguaia River basin is currently undergoing large-scale environmental degradation driven by deforestation for soybean cultivation and extensive cattle ranching (Pelicice et al., 2021), resulting in the mobilization and transport of naturally soil-bound Hg to floodplain lakes (Monteiro et al., 2023). These contrasting environmental settings and disturbance histories provide an appropriate framework for comparing Hg bioaccumulation patterns in fish species that support regional subsistence and commercial fisheries.

Fish consumption is the primary non-occupational exposure pathway for humans (UNEP, 2019), which has motivated national and international regulatory agencies to establish safety limits for Hg concentrations in fish and for weekly Hg intake. In Brazil, the National Health Surveillance Agency (ANVISA) sets maximum permissible concentrations at 500 µg kg^−1^ for non-predatory species and 1000 µg kg^−1^ for predatory species (Brazil, 2013). Internationally, the Food and Agriculture Organization (FAO) recommends a uniform limit of 500 µg kg^−1^ irrespective of trophic guild (FAO, 2011), and the Joint FAO/WHO Expert Committee on Food Additives (JECFA) established a provisional tolerable weekly intake of 1.6 μg kg^−1^ of body weight (JECFA, 2011). Fish consumption is an important source of protein for riverine populations in the Amazon and Araguaia River basins (Braudes-Araújo et al., 2016; Begossi et al., 2018). Thus, even when fish Hg concentrations comply with regulatory thresholds, frequent consumption can still result in elevated exposure among riverine populations, often exceeding tolerable intake levels (Soares et al., 2018; Silva & Oliveira Lima, 2020; Basta et al., 2023; Canela et al., 2024; Cabral et al., 2025). Therefore, evaluating Hg concentrations in fish consumed by local communities is essential for assessing potential human exposure, particularly when considering site-specific consumption patterns and environmental scenarios (Paiva et al., 2025).

In this study, we evaluated Hg accumulation in fish from floodplain lakes hydrologically connected to two major river systems in Brazil, the Madeira River (Amazonia) and the Araguaia River (Cerrado), which differ markedly in their environmental conditions and dominant sources of Hg. Specifically, we aimed to quantify total Hg (THg) concentrations across multiple trophic guilds and evaluate differences in biomagnification patterns between basins, and to assess human exposure to Hg through fish consumption by estimating the weekly intake (EWI) under three realistic consumption scenarios. We hypothesized higher Hg accumulation in fish from the Madeira River basin. In the Amazon basin, Hg inputs are driven by the combined effects of deforestation and ASGM, whereas in the Cerrado, Hg inputs are primarily associated with deforestation and land-use change. We further expected Hg concentrations to increase with trophic level in both basins, leading to higher human exposure through the consumption of carnivorous and piscivorous species, particularly under scenarios representative of traditional riverine populations. The integration of ecological and public health perspectives within this comparative framework provides a robust basis for understanding Hg risks and guiding management strategies tailored to representative Amazonian and Cerrado river systems.

## Materials and methods

### Study area

**Fig. 1:**
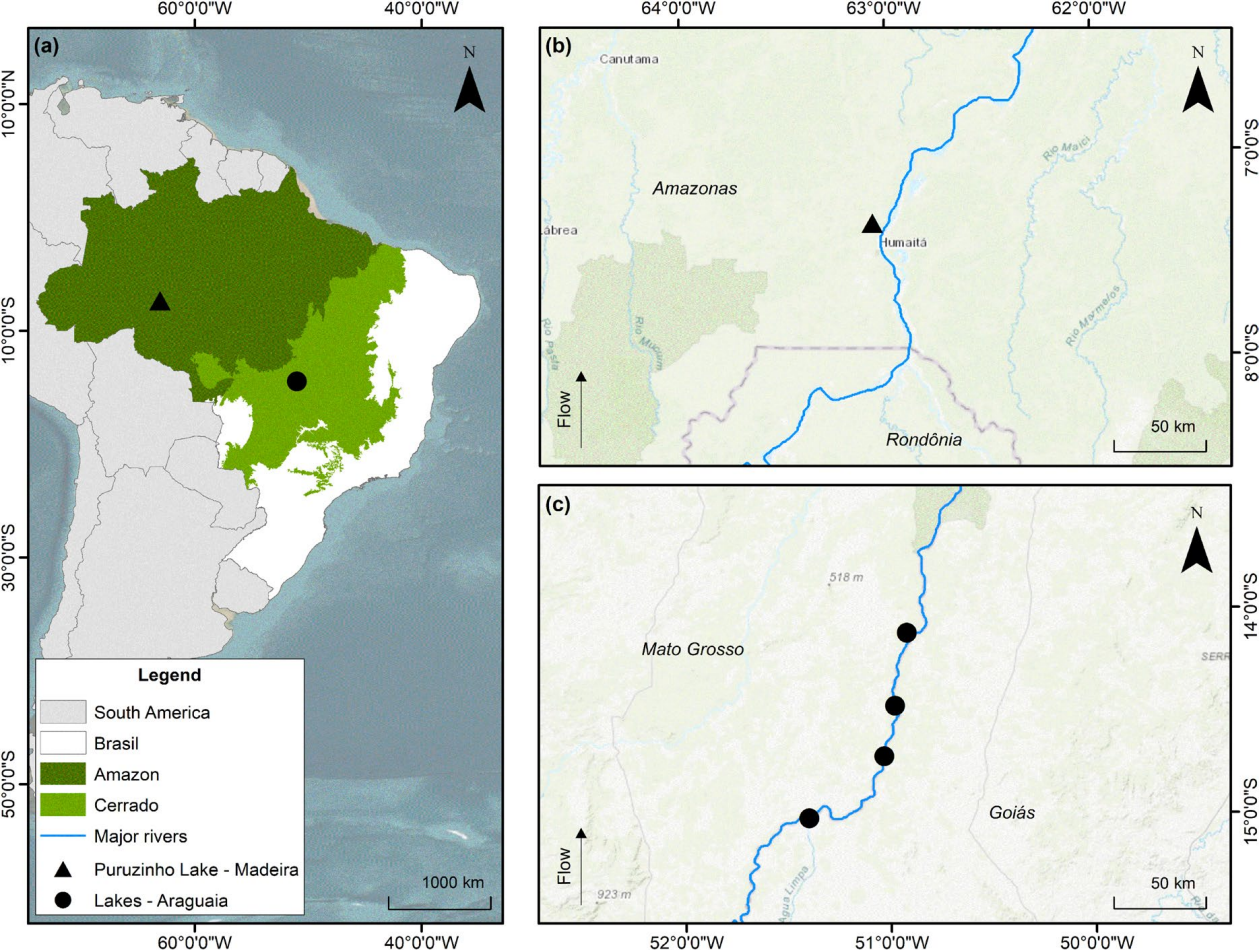
Location of the study areas in relation to Brazil and South America (**a**), highlighting Lake Puruzinho (**b**) and the lakes of the Araguaia River floodplain (**c**)

Our study was conducted in floodplain lakes connected to the Madeira and Araguaia rivers (Fig. 1). Both study areas have a monomodal hydrological cycle with four well-defined seasonal periods: high water, falling water, low water, and rising water (Junk et al., 2014). The Madeira River basin, located in the Western Amazon, covers approximately 1.4 million km^2^ and drains Brazil, Bolivia, and Peru, with a mean annual discharge of 17,620 m^3^ s^−1^ in its Brazilian section (Dória et al., 2025). The geology of Lake Puruzinho, the study area in the lower Madeira River basin, is mainly characterized by Quaternary sedimentary formations, which are part of the Western Amazonian Low Plateau (Almeida, 2006; Tapias et al., 2019).

The Araguaia River is a large free-flowing river predominantly located within the Cerrado biome in central Brazil, draining the states of Goiás, Mato Grosso, Tocantins, and Pará over an area of approximately 377,000 km^2^. In its middle section, which is the focus of this study, the river exhibits a mean annual discharge of about 6420 m^3^ s^−1^ (Aquino et al., 2009). The Middle Araguaia is mainly composed of Quaternary terraces and alluvial deposits of the Araguaia Formation (CPRM, 2004). The geomorphic setting and the low topographic gradients favor the development of extensive floodplains and encompass a complex mosaic of nearly 300 floodplain lakes, as well as Bananal Island, the largest fluvial island in the world (Morais et al., 2005; Pelicice et al., 2025).

### Sampling design

Fish sampling yielded 352 individuals representing 19 species from the Madeira River basin (Lake Puruzinho). In the Araguaia River floodplain, 274 individuals of 17 species were collected from four lakes. The species were classified into trophic guilds based on information from the FishBase platform (Froese & Pauly, 2025) and ichthyological guides for each river basin (Venere & Garutti, 2011; Cella-Ribeiro et al., 2016). In Lake Puruzinho, the assemblage comprised 2 herbivores, 103 detritivores, 3 omnivores, 6 planktivores, 101 carnivores, and 137 piscivores. In the Araguaia floodplain lakes, 11 herbivores, 148 detritivores, 32 omnivores, 7 planktivores, 47 carnivores, and 29 piscivores were analyzed. Fish were collected using nets with mesh sizes ranging from 3 to 16 cm between knots to ensure representative sampling across different size classes and trophic guilds. All individuals were evaluated for total weight and standard length. An aliquot of the dorsal muscle of each specimen was collected with a scalpel and frozen until THg determination.

Sampling campaigns were conducted in both study areas between 2019 and 2024, covering three hydrological periods: rising water (RW), high water (HW), and low water (LW). In the Madeira River basin, collections were conducted in December 2019 (RW), May 2023 (HW), March 2024 (HW), and October 2024 (LW). In the Araguaia River basin, campaigns were carried out in November 2021 (RW), February 2022 (HW), February 2023 (HW), and September 2024 (LW). This sampling design was structured to capture variation in Hg concentrations at the community level throughout the entire hydrological cycle; however, the imbalance in the number of samples across trophic guilds between hydrological periods precludes statistical inferences about seasonal dynamics. The collections were authorized by the Chico Mendes Institute for Biodiversity Conservation (SISBIO no. 66,019-12 to 15 and 65,585-9).

### Total mercury determination

Samples of dorsal muscle were solubilized with 0.5 mL of hydrogen peroxide (H_2_O_2_ 30%, m/v) and 4 mL of a mixture of sulfuric acid and nitric acid (H_2_SO_4_:HNO_3_, 1:1) in a digestion block for 30 min at 70 °C. Next, 5 mL of potassium permanganate (KMnO_4_ 5%, m/v) was added, and the samples were returned to the digestion block for 20 min. The samples were kept overnight and titrated with 0.5 mL of hydroxylamine hydrochloride (NH_2_OH HCl 12%, m/v). The final volume of 14 mL was completed with ultrapure water (Milli-Q Plus, Millipore, Bedford, USA). THg quantification was performed by cold vapor atomic absorption spectrometry (CVAAS) using FIMS-400 equipment (Perkin Elmer, Norwalk, USA). The mean limit of detection was 5 ± 3 µg kg^−1^. The accuracy of the analytical method was evaluated using the certified reference materials DORM-2 (Dogfish muscle, National Research Council, Ottawa, Canada) and IAEA-436 (Tuna fish, International Atomic Energy Agency, Brussels, Belgium), with recovery rates between 89 and 120% (n = 16). Duplicate reagent blanks were analyzed at the beginning of each batch, with Hg concentrations ranging from 0.001 to 0.112 µg L^−1^ (0.055 ± 0.041 µg L^−1^, n = 16). The analyses were performed at the Environmental Biogeochemistry Laboratory—Wolfgang C. Pfeiffer at the Federal University of Rondônia. All concentrations were reported on a wet weight (ww) basis.

### Estimates of mercury intake by humans through fish consumption

The concentrations of THg determined in fish muscle were compared with the safety limits established by Brazilian legislation, 500 µg kg^−1^ for non-predatory species and 1000 µg kg^−1^ for predatory species (Brazil, 2013). Estimates of weekly Hg intake (EWI) through fish consumption were calculated based on the concentration of THg in each specimen (µg kg^−1^), the average body weight of the human population (kg), and weekly fish consumption (kg week^−1^). A body weight of 65 kg was adopted for both river basins, based on the medians for the North and Central-West regions of Brazil (IBGE, 2008). THg concentrations were adjusted using a conversion factor of 0.8 to estimate MeHg concentrations indirectly. This more conservative approach was adopted instead of assuming MeHg proportions of 90–100% (Soares et al., 2018; Hacon et al., 2020; Vasconcellos et al., 2021), thereby avoiding potential overestimation of MeHg concentrations while reflecting the mean MeHg proportions previously determined for fish species from different trophic levels in the Madeira River basin (Bastos et al., 2015; Mussy et al., 2022). The estimates were compared with the provisional upper tolerable weekly intakes of MeHg (PTWI: 1.6 µg kg^−1^ bw week^−1^), established by the Joint FAO/WHO Expert Committee on Food Additives (JECFA) (FAO/WHO, 2003).

The EWI was calculated based on three fish consumption scenarios. Scenarios I and II were based on the national consumption rates for urban (50 g week^−1^) and rural (100 g week^−1^) populations (IBGE, 2020). Scenario III was adopted to represent riverine and indigenous communities, in which fish is the main source of protein. A systematic review on fish consumption patterns in the Amazon revealed that traditional communities consume three to six times more fish than urban populations (Miranda, 2024). Moreover, populations living in remote areas may consume up to nine times more fish than those living near urban centers (Miranda, 2024). Therefore, considering the annual per capita consumption of urban populations (50 g week^−1^), we adopted a conservative consumption rate of 300 g week^−1^. EWI values were compared with the PTWI using one-sample t-tests.

### Data analysis

A generalized additive mixed model (GAMM) was used to compare THg concentrations among river basins (Madeira and Araguaia) and trophic guilds (detritivore, planktivore, carnivore, and piscivore), including their interaction and standard length as fixed effects. Samples from the four lakes within the Araguaia basin were analyzed as a single pooled dataset. Species identity was included as a random intercept to account for interspecific variability. Additionally, a GAMM was performed to compare THg concentrations normalized by fish standard length between river basins, following the approach recommended by Eikenberry et al. (2015) for comparing Hg bioaccumulation in fish across different locations and time periods. The models were fitted using restricted maximum likelihood (REML) and assumed a Gamma error distribution with a log link function. Trophic guilds with insufficient sample sizes (herbivores and omnivores) were excluded from the analyses.

The significance of fixed effects and their interaction was assessed using likelihood-based tests. Pairwise comparisons among trophic guilds within each river basin and between river basins within each trophic guild were performed using Wald contrasts on the fixed-effect coefficients derived from the GAMM. Contrasts were computed on the log scale, and p-values were adjusted for multiple comparisons using the Holm correction. The overall significance of the models was tested using a likelihood ratio test, comparing the full models with a null model including only the intercept and the same random effect structure. The proportion of variance explained by the model was quantified using marginal (R^2^_m_) and conditional (R^2^_c_) coefficients of determination, representing the variance explained by fixed effects alone and by both fixed and random effects, respectively.

The trophic magnification slope (TMS) was calculated using simple linear regression between log-normalized THg concentrations in fish and the mean trophic level of each species, where a slope (*b*) significantly greater than zero indicates biomagnification along the trophic chain (Lavoie et al., 2013). Trophic levels were obtained from the FishBase platform (Froese & Pauly, 2025). Due to the absence of dietary data for *Agoniates halecinus* in FishBase and the inaccuracy of trophic level estimates based on body size and related taxa, the trophic position of this species was obtained from Keppeler (2015) using stable carbon and nitrogen isotope analysis. Differences in TMS between basins were assessed using analysis of covariance (ANCOVA). Statistical significance was evaluated using Monte Carlo permutation tests (9999 permutations) based on the Freedman-Lane procedure. Additionally, samples from each study area were ranked according to their THg concentrations and converted into one-dimensional Euclidean distances to assess the relative length of the food chains based on Hg bioaccumulation, following the approach proposed by Hernández-Arciga et al. (2016) to evaluate isotopic distances. The Euclidean distances in the two study areas were compared using the Mann–Whitney test. All analyses were performed in R (R Core Team, 2025), using the packages mgcv (Wood, 2025), MuMIn (Barton, 2025), permuco (Frossard & Renaud, 2024).

## Results

### Comparison of mercury concentrations between river basins

Total Hg concentrations in fish from the Madeira River ranged from 37 to 2,473 µg kg^−1^ (mean ± standard deviation: 750 ± 516 µg kg^−1^) (Table S1). Concentrations above the safety limits for predatory (1,000 µg kg^−1^) and non-predatory species (500 µg kg^−1^) were determined in 35.5% of the specimens (n = 125), mainly the carnivorous species *Cichla pleiozona* (n = 41) and the piscivorous species *Serrasalmus rhombeus* and *Pellona castelnaeana* (n = 25 each). Among non-predatory species, all individuals of the planktivorous species *Hypophthalmus marginatus* showed concentrations close to or above the reference threshold (484–907 µg kg^−1^). In the Araguaia River floodplain lakes, THg concentrations in fish ranged from 6 to 1,947 µg kg^−1^ (202 ± 295 µg kg^−1^), with only 11 samples exceeding safety limits (4%): *Agoniates halecinus* (n = 3), *Anodus orinocencis* (n = 3), *Pellona castelnaeana* (n = 3), *Ageneiosus inermis* (n = 1), and *Rhaphiodon vulpinus* (n = 1) (Table S1).

**Fig. 2:**
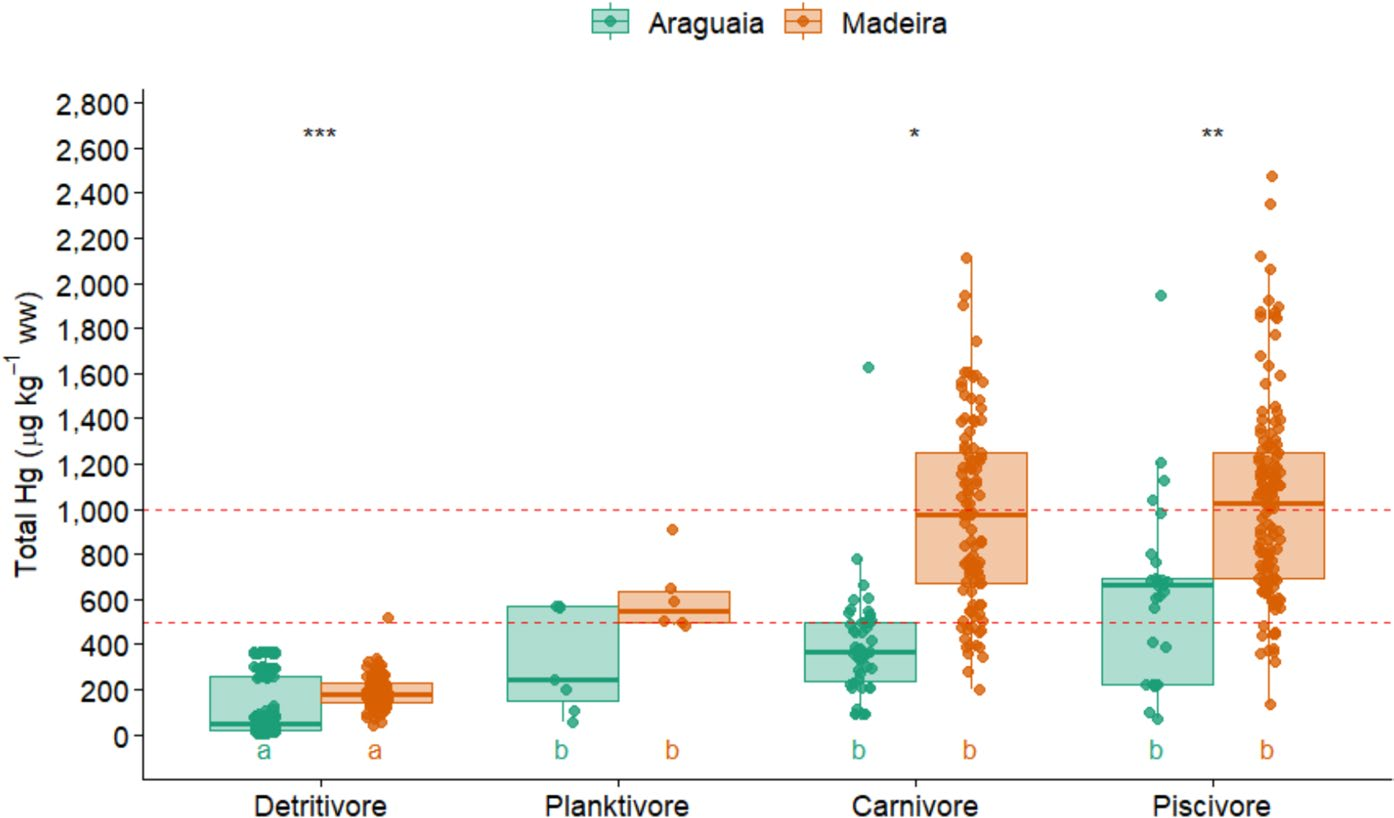
Total Hg concentrations (µg kg^−1^ ww) in fish trophic guilds from the Araguaia River and the Madeira River basin lakes. Different lowercase letters indicate significant differences among trophic guilds within the same river basin based on post hoc Wald contrasts (p < 0.05). Asterisks denote significant differences between river basins within the same trophic guild, with p < 0.05 (*), p < 0.001 (**), and p < 0.0001 (***). Red dashed lines indicate safety limits for non-predatory (500 µg kg^−1^) and predatory species (1,000 µg kg^−1^). Herbivores and omnivores were not compared due to the small number of samples (n ≤ 3)

The generalized additive mixed model explained a large proportion of the variance in THg concentrations (R^2^_m_ = 0.806, R^2^_c_ = 0.877, p < 0.0001). THg concentrations differed significantly between river basins (F = 27.158, p < 0.0001) and among trophic guilds (F = 33.603, p < 0.0001), with no significant interaction between these factors (p > 0.05). Standard length had a positive effect on THg concentrations (F = 13.540, p = 0.0002). Comparisons between river basins revealed significantly higher THg concentrations in the Madeira River basin for detritivorous (p < 0.0001), carnivorous (p = 0.045), and piscivorous fish (p = 0.008), whereas no significant difference was observed for planktivorous species (p > 0.05) (Fig. 2; Table S1). When normalized by fish standard length, THg concentrations also differed significantly between river basins (F = 22.07, p < 0.0001), but only for detritivorous species (p = 0.0001; Figure S1) (R^2^_m_ = 0.699; R^2^_c_ = 0.801, p < 0.0001).

In the Madeira River basin, THg concentrations in piscivorous (1,033 ± 431 µg kg^−1^, p < 0.0001), carnivorous (976 ± 415 µg kg^−1^, p < 0.0001), and planktivores guilds (606 ± 161 µg kg^−1^, p = 0.019) were significantly higher than those determined in detritivorous species (189 ± 72 µg kg^−1^) (Fig. 2). The same pattern was observed in the Araguaia River basin, with significantly higher concentrations in piscivorous (744 ± 355 µg kg^−1^, p < 0.0001), carnivorous, (465 ± 263 µg kg^−1^, p < 0.0001) and planktivorous species (328 ± 233 µg kg^−1^, p = 0.0001) compared to detritivorous species (42 ± 39 µg kg^−1^) (Fig. 2). Herbivorous and omnivorous species were not statistically compared due to the small sample size in the Madeira River basin. In the Madeira basin, THg concentrations were 125 ± 76 µg kg^−1^ in herbivorous species and 171 ± 196 µg kg^−1^ in omnivorous species. In the Araguaia basin, herbivorous species exhibited lower THg concentrations (79 ± 75 µg kg^−1^) than omnivorous species (328 ± 233 µg kg^−1^).

### Biomagnification of mercury along food chains

**Fig. 3:**
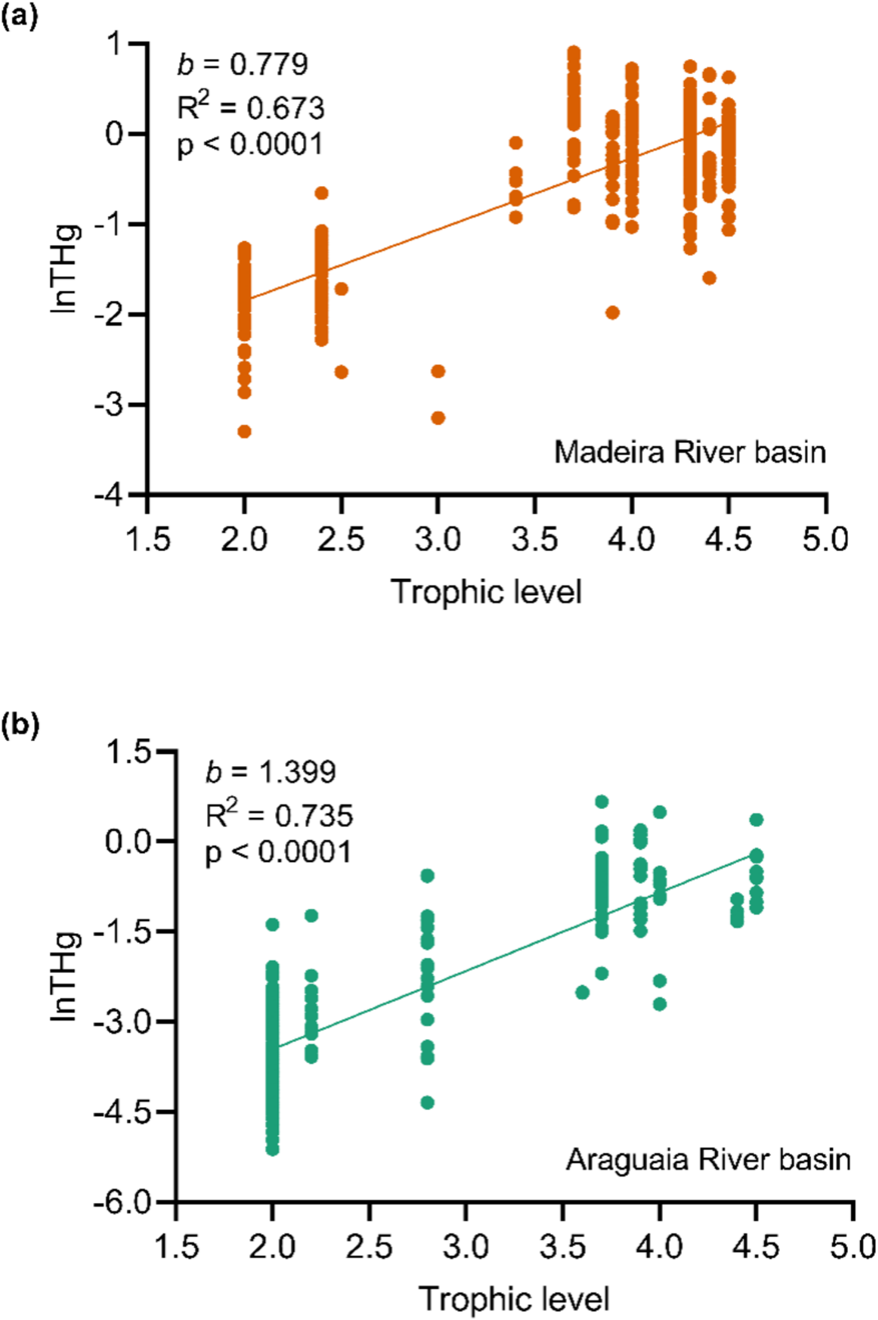
Relationship between mean trophic level and total mercury concentrations, expressed as natural logarithm (ln THg), in (**a**) Puruzinho Lake, Madeira River basin, and (**b**) Araguaia River basin. Trophic levels were obtained from the FishBase platform (Froese & Pauly, 2025), except for *Agoniates halecinus* (Keppeler, 2015)

Trophic magnification slopes (TMS) confirmed biomagnification in both study areas, with significantly higher slopes in the Araguaia River basin (ANCOVA: F = 115.7, p = 0.0001). The slope was 1.399 (95% CI 1.290–1.508) in the Araguaia basin (R^2^ = 0.735, p < 0.0001) (Fig. 3b) and 0.779 (95% CI 0.722–0.837) in the Madeira basin (R^2^ = 0.673, p < 0.0001) (Fig. 3a). However, the one-dimensional Euclidean distance indicated a greater trophic breadth in the Madeira River basin (U = 17,220, p < 0.0001), with a distance of 5.9 between the detritivorous species *Hemiodus unimaculatus* and the piscivorous species *Pellona castelnaeana*. In contrast, a shorter distance (3.8) was observed in the Araguaia River basin between the detritivorous species *Psectrogaster amazonica* and *Pellona castelnaeana*.

### Estimates of Hg intake through fish consumption

Hg intake estimates were substantially higher in the Madeira River basin, reaching 0.425 ± 0.294 µg kg^−1^ bw week^−1^ for scenario I (50 g week^−1^), 0.896 ± 0.617 µg kg^−1^ bw week^−1^ for scenario II (100 g week^−1^), and 2.769 ± 1.907 µg kg^−1^ bw week^−1^ for scenario III (300 g week^−1^) (Table 1). In scenario II, 45 individuals belonging to piscivorous and carnivorous species showed EWI values exceeding the safety threshold. In scenario III, representative of traditional populations, 230 samples exceeded the PTWI, including one individual of the detritivorous species *Prochilodus nigricans* and six of the planktivorous species *Hypophthalmus marginatus*. The species *Cichla pleiozona*, *Serrasalmus rhombeus*, *Pellona castelnaeana*, and *Pinirampus pirinampu* stood out as the main potential dietary sources of Hg exposure to humans.

**Table 1:** Estimated weekly intake of total mercury (EWI, µg kg^−1^ bw week^−1^) by trophic guild and river basin under three fish consumption scenarios: scenario I (Urban, 50 g week^−1^), scenario II (Rural, 100 g week^−1^), and scenario III (Riverine, 300 g week^−1^)

River basin	Trophic guild	Urban (50 g week^−1^)		Rural (100 g week^−1^)		Riverine (300 g week^−1^)	
		EWI	> PTWI	EWI	> PTWI	EWI	> PTWI
Madeira	Herbivore (2)	0.071 ± 0.043 (0.040–0.101)	0 (0)	0.149 ± 0.091 (0.085–0.214)	0 (0)	0.462 ± 0.281 (0.263–0.661)	0 (0)
	Detritivore (103)	0.107 ± 0.041 (0.021–0.294)	0 (0)	0.226 ± 0.087 (0.044–0.621)	0 (0)	0.700 ± 0.268 (0.137–1.919)	0.9 (1)
	Omnivore (3)	0.097 ± 0.111 (0.024–0.225)	0 (0)	0.204 ± 0.235 (0.051–0.474)	0 (0)	0.630 ± 0.726 (0.159–1.466)	0 (0)
	Planktivore (6)	0.343 ± 0.091 (0.274–0.514)	0 (0)	0.723 ± 0.192 (0.578–1.083)	0 (0)	2.237 ± 0.593* **(1.787–3.349)**	100 (6)
	Carnivore (101)	0.552 ± 0.235 (0.115–1.195)	0 (0)	1.165 ± 0.496 (0.242– 2.519)	20.8 (21)	3.603 ± 1.533* **(0.748–7.791)**	91.1 (92)
	Piscivore (137)	0.586 ± 0.247 (0.078–1.522)	0 (0)	1.234 ± 0.517 (0.165–3.043)	17.5 (24)	3.815 ± 1.590* **(0.510–9.130)**	95.6 (131)
Araguaia	Herbivore (11)	0.049 ± 0.046 (0.017–0.180)	0 (0)	0.097 ± 0.092 (0.034–0.359)	0 (0)	0.291 ± 0.275 (0.103–1.078)	0 (0)
	Detritivore (148)	0.026 ± 0.024 (0.004–0.155)	0 (0)	0.052 ± 0.048 (0.007–0.310)	0 (0)	0.156 ± 0.145 (0.022–0.930)	0 (0)
	Omnivore (32)	0.048 ± 0.041 (0.008–0.178)	0 (0)	0.096 ± 0.081 (0.016–0.356)	0 (0)	0.289 ± 0.243 (0.048–1.067)	0 (0)
	Planktivore (7)	0.202 ± 0.143 (0.032–0.352)	0 (0)	0.404 ± 0.287 (0.064–0.704)	0 (0)	1.213 ± 0.861 (0.192–2.112)	42.8 (3)
	Carnivore (47)	0.286 ± 0.162 (0.069– 1.004)	0 (0)	0.572 ± 0.323 (0.138–2.007)	4.2 (2)	1.717 ± 0.970 (0.414–6.022)	46.8 (22)
	Piscivore (29)	0.458 ± 0.219 (0.041–1.198)	0 (0)	0.916 ± 0.438 (0.082–2.396)	3.4 (1)	2.748 ± 1.313* **(0.247–7.189)**	86.2 (25)

A body weight of 65 kg was adopted for both river basins, based on the medians for the North and Central-West regions of Brazil (IBGE, 2008). Values are presented as mean ± standard deviation, with minimum and maximum values in parentheses. The proportion of individuals exceeding the provisional tolerable weekly intake (> PTWI) is expressed as a percentage (%), with the sample size indicated in parentheses (n)

Concentrations shown in bold and marked with an asterisk indicate that the EWI significantly exceeded the PTWI, based on p-values < 0.05 and confidence intervals entirely above 1.6 µg kg^−1^ bw week^−1^

In the Araguaia River basin, weekly Hg intake estimates were 0.124 ± 0.182 µg kg^−1^ bw week^−1^ for scenario I, 0.249 ± 0.363 µg kg^−1^ bw week^−1^ for scenario II, and 0.746 ± 1.090 µg kg^−1^ bw week^−1^ for scenario III (Table 1). No species exceeded the PTWI in scenario I, whereas individuals of *Pellona castelnaeana* (2.396 µg kg^−1^ bw week^−1^), *Ageneiosus inermis* (2.007 µg kg^−1^ bw week^−1^), and *Rhaphiodon vulpinus* (1.770 µg kg^−1^ bw week^−1^) exceeded the safety threshold in scenario II. In scenario III, 50 individuals exceeded the PTWI, including the planktivorous species *Anodus orinocensis* and the predatory species *Agoniates halecinus*, *Ageneiosus inermis, Pellona castelnaeana*, *Pygocentrus nattereri*, and *Rhaphiodon vulpinus*.

Significant exceedances of the PTWI were restricted to riverine consumption patterns (scenario III). In the Madeira basin, riverine communities consuming planktivorous (p = 0.0466; CI = 1.61–2.86 µg kg^−1^ bw week^−1^), piscivorous (p < 0.0001; CI = 3.55–4.08 µg kg^−1^ bw week^−1^), and carnivorous fish (p < 0.0001, CI = 3.30–3.91 µg kg^−1^ bw week^−1^) exhibited EWI values significantly higher than the PTWI. In the Araguaia basin, this pattern was observed only for riverine populations consuming piscivorous species (p < 0.0001; CI = 2.25–3.25 µg kg^−1^ bw week^−1^). No significant exceedance of the PTWI was detected for urban or rural consumption scenarios in either basin. Considering the three exposure scenarios, herbivorous, detritivorous, and omnivorous species represent the safest options for human consumption in both river basins.

## Discussion

THg concentrations were significantly higher in Lake Puruzinho (Madeira river basin) across all trophic guilds, reflecting contrasting Hg sources and biogeochemical processes in the two study areas. In the Madeira Basin, elevated THg burdens are largely attributed to the long history of ASGM, which released substantial quantities of metallic Hg into aquatic systems over the past five decades (Lacerda et al., 2024; Reuther, 1994). ASGM peaked during the 1970s and 1980s as gold prices increased and extraction practices rapidly expanded (Balzino et al., 2015). Although mining intensity has declined, Hg stored in forest soils continues to be remobilized following land-use conversion, providing a sustained secondary source of inorganic Hg to the river system (Lacerda et al., 2012). Lacerda et al. (2024) documented the combined influence of historical ASGM and land-use change on long-term Hg trends, reporting significant increases in THg for *Cichla pleiozona* (carnivore) and *Prochilodus nigricans* (detritivore) in the Upper Madeira River between 2002 and 2021. It should be noted that, in our study, the comparison was performed exclusively among lakes, where fish exhibited significantly higher concentrations than those determined in the main channel of the Madeira River (Lacerda et al., 2024).

Small-scale and intermittent gold mining also occurred in some Araguaia tributaries during the eighteenth century and again in the late twentieth century (Ulrich et al., 2021), but these activities were geographically distant from our sampling sites and did not result in detectable Hg enrichment in nearby floodplain lakes. Lakes downstream of historical mining zones in the Vermelho and Crixás sub-basins exhibit sediment THg concentrations from 28 to 63 µg kg^−1^ (Monteiro et al., 2024), values that fall within background ranges for non-impacted neotropical lakes (Leady & Gottgens, 2001; Monteiro et al., 2023; Escobar-Camacho et al., 2024). By comparison, sediments directly influenced by ASGM exceed 500 µg kg^−1^ in the Beni River, a major tributary of Madeira River (Quintarelli et al., 2024). These contrasts support the conclusion that historical mining does not constitute a significant source of Hg to the Araguaia floodplain lakes and instead highlight land-use change associated with agriculture and cattle ranching as the primary anthropogenic pressure in the basin.

The main channel of the Araguaia River remains free-flowing, unlike several other Brazilian rivers affected by dam construction, such as the Madeira (Bastos et al., 2020; Kasper et al., 2012), Tocantins (Paiva et al., 2024), Teles Pires (Oliveira et al., 2025), and Uatumã rivers (Kasper et al., 2014). In the Madeira River basin, two run-of-river hydropower plants have modified water residence time, sediment transport, and redox conditions in surrounding areas of Lake Puruzinho, all of which can enhance MeHg production, as widely documented in dam-affected Amazonian systems (Pestana et al., 2019). In the Tocantins River basin, considered the “half-sibling” of the Araguaia River, the construction of the Tucuruí Hydropower Plant led to an increased bioaccumulation in fish in the last 35 years, particularly in the omnivorous and planktivorous guilds (Paiva et al., 2024). This increase has been attributed to the combined effects of impoundment, recent land-use changes, and fire occurrence in the region, which may alter biogeochemical processes and food-web structure (Paiva et al., 2024). This mechanism is particularly relevant for migratory fish that move between the main channel and floodplain lakes, exposing them to distinct food sources and spatial MeHg gradients (Azevedo-Silva et al., 2016). In our dataset, *Pellona castelnaeana* exhibits long-distance migration, whereas *Rhaphiodon vulpinus*, *Ageneiosus inermis*, *Psectrogaster amazonica*, and *Triportheus* spp. display intermediate mobility patterns (Campos et al., 2025), which may partially explain species-specific differences in Hg accumulation.

Despite the marked differences in Hg bioaccumulation in fish between the two basins, THg concentrations in abiotic and biotic matrices remain comparable. In bottom sediments from Lake Puruzinho, THg concentrations ranged from 44 to 129 µg kg^−1^ in the dry season and from 32 to 146 µg kg^−1^ in the rainy season (Almeida et al., 2014). Similarly, in lakes associated with the Araguaia River, values ranged from 23 to 82 µg kg^−1^ during the dry season (Monteiro et al., 2023) and 10 to 107 µg kg^−1^ during the rainy season (Monteiro et al., 2024). Comparable values were also observed in plankton: in the Samuel Reservoir (Madeira Basin), THg in phyto- and zooplankton ranged from 4 to 232 µg kg^−1^ (Nascimento et al., 2020), within the same range reported for the Araguaia Basin (16–321 µg kg^−1^) (Monteiro et al., 2024). A similar pattern occurs for macrophytes. Pestana et al. (2016) reported concentrations between 23 and 58 µg kg^−1^ for rooted (*Oryza* spp.) and floating (*Pontederia crassipes*) species in the Madeira Basin, whereas in the Araguaia Basin values ranged from 5 to 77 µg kg^−1^, including *Paspalum repens*, *Pontederia azurea*, *Pontederia crassipes* and *Salvinia* spp. (Monteiro et al., 2024).

A clear biomagnification pattern was observed in both river basins (TMS > 0), although it was significantly stronger in the Araguaia basin, indicating more efficient Hg transfer through the aquatic food web. In contrast, the Madeira basin exhibited a broader trophic range, suggesting greater food web complexity despite a lower trophic magnification slope. Notably, THg concentrations normalized by fish size were higher in the Madeira basin only for the detritivorous guild. This pattern indicates more efficient Hg accumulation at the base of the food web and, consequently, greater Hg availability for higher trophic levels, which may help explain the broader trophic range identified by the univariate Euclidean distance. In addition to historical Hg emissions in the Madeira River basin, the observed differences likely reflect contrasting environmental conditions in floodplain lakes, including seasonal hydrological dynamics that influence carbon sources and trophic structure (Pouilly et al., 2013; Mussy et al., 2022).

Variations in foraging behavior within trophic guilds may also contribute to these patterns. For instance, the planktivorous species *Hypophthalmus marginatus* from the Madeira basin primarily feeds on zooplankton (Cella-Ribeiro et al., 2016). Zooplankton organisms exhibited high Hg concentrations (15–861 μg kg-1), with MeHg accounting for up to 100% of total Hg (Oliveira et al., 2026), representing an important pathway for Hg transfer to higher trophic levels. In contrast, *Anodus orinocensis*, collected in the Araguaia basin, feeds mainly on periphytic algae (Cella-Ribeiro et al., 2016), which are recognized as hotspots of MeHg production in Neotropical floodplains (Coelho-Souza et al., 2011; Molina et al., 2010). Therefore, despite the clear trophic patterns observed within each study area, the lack of species standardization between basins may contribute to contrasting biomagnification patterns.

The highest THg concentrations in both study areas were recorded in *Pellona castelnaeana*. This species exhibits fast predatory strikes in surface and mid-water layers and has a strictly piscivorous diet (Cella-Ribeiro et al., 2016), resulting in pronounced Hg bioaccumulation throughout its lifespan. Therefore, *Pellona castelnaeana* serves as an effective bioindicator of trophic transfer of Hg in Neotropical aquatic ecosystems. *Hemiodus unimaculatus* and *Psectrogaster amazonica*, in contrast, occupy the base of the trophic networks in both the Madeira and Araguaia River basins. Both species are detritivores that feed on organic matter and sediment-associated resources (Cella-Ribeiro et al., 2016), which restricts Hg uptake and results in relatively low THg concentrations. Despite the low bioaccumulation in lower-trophic-level species, pronounced biomagnification through the food web can affect human health via dietary exposure to predatory fish.

Although no recent data are available on fish consumption by riverine communities in the Araguaia River, a study conducted in the late 1990s showed that fish accounted for only 10% of the protein intake of the local population, unlike riverine communities in the Amazon basin (Begossi et al., 2000). Preliminary results from our research group indicate that individuals who consume fish rarely and those who consume it weekly represent similar proportions of the population, whereas smaller proportions report monthly or daily consumption. These patterns suggest that fish consumption in the Araguaia Basin is highly heterogeneous and varies according to the degree of economic dependence on the river. A recent study reported that among 75 artisanal fishers in the Middle Araguaia, 85% consumed fish three to seven times per week (Mendes Filho et al., 2020). Silva et al., (2019a, 2019b) also highlighted that fish remains an important protein source for the Iny Karajá indigenous communities residing in the Middle Araguaia. In this context, Hg intake estimates indicate that low (50 g week^−1^) and moderate (100 g week^−1^) fish consumption pose little risk to human health. Conversely, for populations consuming higher quantities of fish (300 g week^−1^), such as artisanal fishers and indigenous communities, consumption of carnivorous and piscivorous species represents a potential source of Hg exposure.

In the Madeira River basin, by contrast, the moderate consumption of carnivorous and piscivorous species may pose potential health risks, and the weekly intake of 300 g of planktivorous, carnivorous, or piscivorous fish exceeded the Hg safety threshold. In both basins, herbivorous, detritivorous, and omnivorous species presented low human health risks. In addition to the relatively low Hg concentrations observed in our study, these species are highly nutritious (Heilpern et al., 2025) and contain elevated selenium levels, which can mitigate Hg toxicity by forming stable Hg–Se complexes that reduce its bioavailability (Cabral et al., 2025). However, fish is the primary source of protein for riverine communities in the Madeira Basin, with consumption rates ranging from 320 to 406 g per day (Oliveira et al., 2010; Dória et al., 2016). Most individuals consume fish 3–7 days per week, resulting in elevated Hg concentrations in human biomarkers, including among women of reproductive age and children (Canela et al., 2024; Hacon et al., 2014; Marques et al., 2013; Mendes et al., 2021; Oliveira et al., 2010). Therefore, fixed national and international safety limits do not uniformly protect consumers across Neotropical regions, making it necessary for risk assessments to account for region-specific dietary practices and ecological conditions.

## Strengths, limitations, and future perspectives

Our study is closely aligned with the Minamata Convention on Mercury, to which Brazil has been a signatory since 2018, reinforcing the importance of research addressing the impacts of Hg and its compounds on the environment and human health, particularly with regard to vulnerable populations, such as Indigenous and riverine communities exposed to biomagnification and contamination of traditional food sources (i.e., fish consumption) (Brazil, 2018). This assessment is especially relevant within the context of the Araguaia River basin, which encompasses a mosaic of Indigenous territories containing multiple villages of the Iny Karajá, Apyãwa (Tapirapé), Javaé, and Avá-Canoeiro peoples within the area of influence of our study region. Based on a large and taxonomically diverse dataset encompassing multiple trophic levels and seasonal periods, we demonstrate that relatively elevated Hg concentrations and clear biomagnification patterns also occur in the Araguaia River basin, located in the Brazilian Savanna, where land-use change and wildfires are the dominant anthropogenic pressures. In addition to these anthropogenic impacts, several hydropower proposals are currently under evaluation, including projects planned directly on the main channel, although the Araguaia River remains a free-flowing system (Latrubesse et al., 2019). This context reinforces the importance of our baseline assessment for anticipating potential future changes in Hg dynamics.

The low number of shared species between basins represents a major limitation of this study, as it precludes direct species-level comparisons. Although species identity was included as a random effect in the additive models to account for interspecific variability, this approach does not fully overcome the lack of species equivalence between basins. Therefore, some of the observed differences may still reflect variations in species composition. Additionally, although our dataset comprises three hydrological periods (rising water, high water, and low water), the imbalance in the number of samples for each trophic guild across periods precluded the analysis of seasonal bioaccumulation patterns. The lack of concomitant sampling of water, sediment, and basal resource samples may also represent a limitation for comparing Hg contamination between river basins and identifying potential sources of exposure to fish. Nevertheless, the GAMM results and the clear biomagnification patterns reinforce the role of fish as reliable bioindicators of environmental Hg contamination.

Although extensive research conducted in the Madeira Basin provides substantial evidence supporting a better understanding of how anthropogenic impacts influence the biogeochemical cycling of Hg, significant knowledge gaps remain for the Araguaia Basin. Scientific efforts in the region are comparatively recent and limited mainly to environmental samples and total Hg determinations. There is currently no data on Hg exposure in human populations from the Araguaia Basin, which represents a critical gap for risk assessment. Future studies should prioritize: (i) understanding the factors regulating MeHg production in depositional floodplain environments and quantifying its transfer at the base of the food web; (ii) evaluating the effects of the seasonal flood pulse on THg distribution across different environmental matrices, including fish; (iii) assessing the influence of migratory fish species on community structure and Hg biomagnification at the local scale; and (iv) quantifying Hg in human biomarkers and conducting detailed characterizations of riverine diets, including species preferences, seasonal availability, and the contribution of non-fish protein sources.

## Conclusions

Our study demonstrates consistent patterns of Hg biomagnification in freshwater fish communities from two major impacted South American basins. In both systems, Hg concentrations increased from detritivorous to piscivorous species, confirming trophic transfer, while overall concentrations were significantly higher in the Madeira basin, particularly among detritivorous, carnivorous, and piscivorous fish. Trophic magnification slopes revealed contrasting biomagnification dynamics, with a significantly higher TMS in the Araguaia basin, indicating more efficient Hg transfer across trophic levels despite lower overall concentrations. In contrast, the Madeira basin exhibited a broader trophic structure but lower magnification efficiency, suggesting a decoupling between food web structure and contaminant transfer. These results emphasize the need to assess Hg dynamics in basins without a significant mining legacy, as deforestation-driven soil erosion and biomass burning can mobilize Hg and sustain its transfer through aquatic food webs even in the absence of ASGM.

Human exposure assessments showed that Hg intake frequently exceeded safety thresholds under high fish consumption scenarios, particularly in the Madeira basin and among carnivorous and piscivorous species. In the Araguaia basin, exceedances were less frequent but still occurred under riverine consumption patterns, mainly for piscivorous species. Herbivorous and detritivorous species represent safer dietary options in both systems. Given that fish constitutes the primary protein source for riverside communities, especially in the Amazon, estimated Hg intake may pose risks to human health. Current safety limits adopted by ANVISA for Hg concentrations in fish and by FAO/WHO for weekly intake may be sufficient to protect populations with relatively low fish consumption, such as those in the Araguaia basin, but appear inadequate for highly fish-dependent communities in the Madeira basin. This mismatch between regional exposure scenarios and regulatory standards underscores the need for health guidelines tailored to local dietary practices.

## Supplementary Information

Below is the link to the electronic supplementary material.

Supplementary file1 (DOCX 36 KB)

## Data Availability

Data will be made available on request.
