# Supplementary material for: Contrasting anthropogenic drivers of mercury bioaccumulation in fish and associated dietary exposure risks in Amazon and Cerrado floodplain lakes
Source: Environ Geochem Health. 2026 Jul 14;48(10):458. doi: 10.1007/s10653-026-03335-0 (PMC13369633; doi:10.1007/s10653-026-03335-0)
Supplement: Supplementary file 1 — Supplementary file1 (DOCX 36 KB) [file 10653_2026_3335_MOESM1_ESM.docx]

**Table S1.** Descriptive statistics of THg concentrations (µg kg^-1^) in fish collected from lakes in the Araguaia and Madeira river basins, grouped by trophic guild. N: number of samples. SD: standard deviation. Min: Minimum. Max: Maximum. > ANVISA: Number of individuals with THg concentrations above the safety limits established by the Brazilian Health Regulatory Agency (ANVISA), corresponding to 500 µg kg^-1^ for non-predatory species and 1000 µg kg^-1^ for predatory species. “–“ indicates that no sample exceeded the safety limit.

| **Basin** | **Trophic guild** | **N** | **Species** | **Mean** | **SD** | **Min** | **Max** | **> ANVISA** |
| --- | --- | --- | --- | --- | --- | --- | --- | --- |
| Araguaia | Herbivore | 11 | *Laemolyta* cf. *fernandezi* | 79 | 75 | 28 | 292 | - |
| Araguaia | Detritivore | 16 | *Curimata inornata* | 51 | 23 | 22 | 102 | - |
| Araguaia | Detritivore | 47 | *Cyphocharax gouldingi* | 20 | 8 | 7 | 48 | - |
| Araguaia | Detritivore | 10 | *Hemiodus unimaculatus* | 106 | 101 | 26 | 252 | - |
| Araguaia | Detritivore | 75 | *Psectrogaster amazonica* | 45 | 28 | 6 | 124 | - |
| Araguaia | Omnivore | 13 | *Tetragonopterus argenteus* | 81 | 1 | 80 | 82 | - |
| Araguaia | Omnivore | 14 | *Triportheus auritus* | 91 | 93 | 27 | 289 | - |
| Araguaia | Omnivore | 5 | *Triportheus trifurcatus* | 35 | 49 | 13 | 122 | - |
| Araguaia | Planktivore | 7 | *Anodus orinocensis* | 328 | 233 | 52 | 572 | 3^a^ |
| Araguaia | Carnivore | 6 | *Lycengraulis batesii* | 309 | 54 | 226 | 361 | - |
| Araguaia | Carnivore | 4 | *Plagioscion squamosissimus* | 312 | 51 | 266 | 383 | - |
| Araguaia | Carnivore | 25 | *Pygocentrus nattereri* | 427 | 130 | 112 | 666 | - |
| Araguaia | Piscivore | 5 | *Ageneiosus inermis* | 726 | 511 | 386 | 1,631 | 1^b^ |
| Araguaia | Piscivore | 15 | *Agoniates halecinus* | 773 | 205 | 563 | 1,209 | 3^b^ |
| Araguaia | Piscivore | 9 | *Pellona castelnaeana* | 890 | 466 | 385 | 1,947 | 3^b^ |
| Araguaia | Piscivore | 9 | *Rhaphiodon vulpinus* | 650 | 337 | 334 | 1,438 | 1^b^ |
| Araguaia | Piscivore | 3 | *Serrasalmus rhombeus* | 191 | 188 | 67 | 407 | - |
|  |  |  |  |  |  |  |  |  |
| Madeira | Herbivore | 2 | *Schizodon fasciatus* | 125 | 76 | 71 | 179 | - |
| Madeira | Detritivore | 2 | *Hemiodus unimaculatus* | 56 | 27 | 37 | 75 | - |
| Madeira | Detritivore | 30 | *Potamorhina altamazonica* | 161 | 42 | 88 | 283 | - |
| Madeira | Detritivore | 6 | *Potamorhina latior* | 228 | 59 | 126 | 273 | - |
| Madeira | Detritivore | 59 | *Prochilodus nigricans* | 213 | 72 | 102 | 520 | 1^a^ |
| Madeira | Detritivore | 6 | *Psectrogaster amazonica* | 104 | 56 | 57 | 211 | - |
| Madeira | Omnivore | 2 | *Brycon amazonicus* | 58 | 21 | 43 | 72 | - |
| Madeira | Omnivore | 1 | *Triportheus albus* | 397 |  |  |  | - |
| Madeira | Planktivore | 6 | *Hypophthalmus marginatus* | 606 | 161 | 484 | 907 | 4^a^ |
| Madeira | Carnivore | 4 | *Ageneiosus inermis* | 553 | 157 | 425 | 778 | - |
| Madeira | Carnivore | 71 | *Cichla pleiozona* | 1064 | 386 | 281 | 2,110 | 41^b^ |
| Madeira | Carnivore | 9 | *Hoplias malabaricus* | 676 | 282 | 345 | 1,028 | 1^b^ |
| Madeira | Carnivore | 17 | *Plagioscion squamosissimus* | 867 | 488 | 203 | 1,944 | 5^b^ |
| Madeira | Piscivore | 21 | *Acestrorhynchus falcirostris* | 823 | 311 | 138 | 1,215 | 10^b^ |
| Madeira | Piscivore | 34 | *Pellona castelnaeana* | 1325 | 512 | 441 | 2,473 | 25^b^ |
| Madeira | Piscivore | 29 | *Pinirampus pirinampu* | 826 | 238 | 447 | 1,300 | 8^b^ |
| Madeira | Piscivore | 3 | *Pseudoplatystoma punctifer* | 431 | 150 | 322 | 602 |  |
| Madeira | Piscivore | 8 | *Rhaphiodon vulpinus* | 1175 | 334 | 900 | 1,873 | 5^b^ |
| Madeira | Piscivore | 42 | *Serrasalmus rhombeus* | 1061 | 379 | 357 | 2,064 | 25^b^ |

^a^Non-predatory (500 µg kg^-1^); ^b^Predatory (1,000 µg kg^-1^)


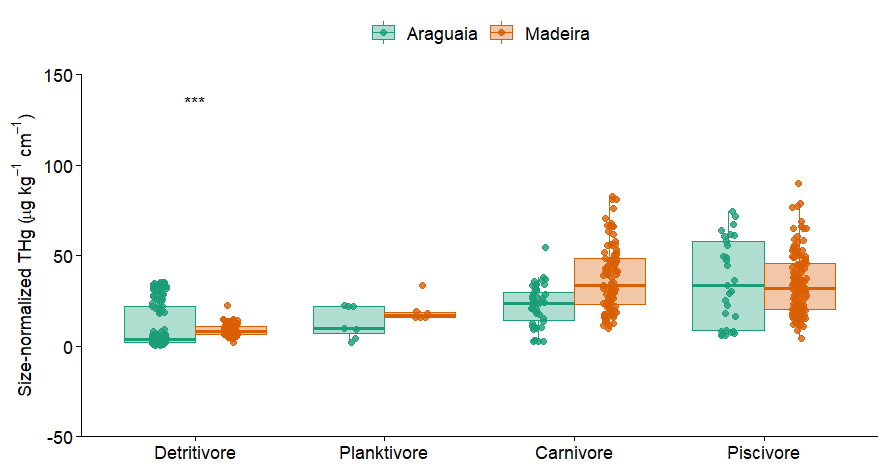


**Figure S1.** Comparison of size-normalized THg concentrations across trophic guilds between river basins. Asterisks indicate a significant difference in the detritivore guild (p < 0.0001), based on Wald contrasts of the fixed-effect coefficients, with p-values adjusted using the Holm correction.
